# Supplementary material for: The impact of atypical early histories on pet or performer chimpanzees
Source: PeerJ. 2014 Sep 23;2:e579. doi: 10.7717/peerj.579 (PMC4179557; doi:10.7717/peerj.579)
Supplement: Supplemental Information 1 — Definitions of 21 behaviors observed in study. [file peerj-02-579-s001.docx]

| **Agonism** |  |
| --- | --- |
| Contact Aggression | Aggressive behaviors that must involve some physical contact between individuals. Includes, wrestling, lunge hit, grab, bite, and scratch. May include pilo-erection. |
| Receive contact aggression | Receiving any of the above behaviors. |
| Non-contact aggression | Aggressive behaviors directed to another individual,that do not include any physical contact. Includes lunge, rush, and threats. Includes charges clearly directed to humans (as indicated byeye contact) when the focal and human target is less than 1 meter from the visitor glass (Modifier:Human) (otherwise-Display) |
| Receive NC aggression | Receiving any of the above non-contact aggression behaviors |
| Display | Aggressive behavior without any clear and identifiable recipient. May include pilo-erection, and such behaviors as beating on or moving inanimate objects, stomping, slapping, swaying, hooting, chest-beat, or running. If these behaviors are clearly directed towards an individual, score as non-contact aggression. |
| Submissive behavior | Includes clear instances of displacement in which the departure of a submissive individual and the arrival of a dominant individual can be reliably connected and occur within a short time window. |

| **Sexual** | |  |
| --- | --- | --- |
| Social Sex | Includes any component of a series of sexual behaviors including mounting, thrusting and complete copulation. May occur with or without full penetration. Includes unsuccessful copulations due to incorrect orientation or unreceptive partner. Includes homosexual mounting. | |
| Masturbation | Actively using a body part, object, or part of the cage to stimulate own genitals, with clear accompanying evidence of pleasure or arousal (bared-tooth grin-like expressions, screams, etc.). Does not include manipulation of the genitals (Sexual Exam) or grooming around the genital area (Solitary-Self). | |
| **Abnormal** |  | |
| Coprophagy | Deliberate ingestion of feces from the self or another individual. Includes instances when the subject catches or holds feces as it is excreted, manipulates feces, or “paints” with feces on an exhibit surface. | |
| Abnormal Plucking | Pulling out own or another animal’s hair; may be ingested. Does not include single hairs pulled out incidentally during self-directed or grooming behaviors. | |
| Abnormal body posturing | Repeated, sustained and purposeless manipulation of a specific area of own body, such as eye-poking, self-patting, ear-covering. | |
| Abnormal Movement | Repeated and sustained movement of body, such as rocking or neck-twisting, with a definitive repetitive pattern. | |

| **Social** |  |
| --- | --- |
| Social Play | Non-aggressive interactions involving two or more animals. Never accompanied by pilo-erection or agonism; may be accompanied by play-face and/or laughing. Includes rough-and-tumble play (fast-paced, vigorous locomotion, wrestling, hitting, pulling, chasing, biting, etc.), quiet play (slower-paced, gentle-tickling, finger and toe manipulation, etc.). May involve the use of an object. |
| Social Groom Give | Picking through hair or at skin of another individual and removing debris with hands and/or mouth. Does not include plucking hair or self-directed behaviors. Includes grooming a conspecific with an object (e.g. comb, stick) |
| Receive social groom | Receiving social groom (see above). If two individuals are mutually grooming, record as Groom. Includes being groomed by a conspecific with an object. |
| Prosocial | Individual interacts with another in a social way that cannot be more specifically classified. The non-focal recipient individual may be non-responsive. If subject is the non-responsive recipient, the behavior is recorded otherwise. Prosocial interactions with “Human” as modifier are limited to begging gestures, active chasing games, or re-assurance seeking from a human, all when both human and ape are within 1 meter of the barrier. Gestures directed to the observer are excluded (instead e.g. Inactive, etc). Does not include attention to a human, a human’s body, or an object held by a human (attention keeper/visitor). |

| **Solitary Behaviors** |  |
| --- | --- |
| Self-groom | Picking through, touching, or rubbing own hair or skin and removing debris with hand, foot and/or mouth. Does not include plucking hair (Pluck) or scratching. Includes behaviors that induce and precede regurgitation that involve self-contact (lip rubbing, etc.). Using an object to touch, groom or rub the body is designated Object-Body. |
| Solitary Play | Individual may play by itself with hands, fingers and toes, other body parts, or use an object as the focus of or tool for play. Typically includes **playful, vigorous and exaggerated movements with no clear purpose**. May be either boisterous or quiet (spinning, somersaulting, vigorous shaking of blankets). |
| Inactive | Individual is not moving and not active in any other behaviors listed. Eyes may be open or closed. Includes instances during which a subject holds or carries an object (including food or water), without actively manipulating it (including chewing or swallowing). |
| Attention | Individual maintains gaze directed towards an individual or an area for longer than 3 seconds while within 1 meter of barrier. |
| Locomotion | Individual changes location in a horizontal or vertical space by moving. |
