# Supplementary material for: The impact of atypical early histories on pet or performer chimpanzees
Source: PeerJ. 2014 Sep 23;2:e579. doi: 10.7717/peerj.579 (PMC4179557; doi:10.7717/peerj.579)
Supplement: Table S1 [file peerj-02-579-s002.docx]

Table S1.

Demographics Information for Subjects

| Focal | | Location | Age at data collection | Sex | Human Exposure  Total days CHI_i =_ 0 | Mixed  Exposure  Total days  CHI_i_ = .5 | Conspecific Exposure  Total days  CHI_i_ = 1 | Average  CHI_i_ value | Categorical Group |
| --- | --- | --- | --- | --- | --- | --- | --- | --- | --- |
| A | Sanctuary1 | | 14 | F | 0 | 1460 | 0 | 0.5 | MIXED |
| B | Sanctuary1 | | 12 | M | 312 | 1148 | 0 | 0.393 | MIXED |
| C | Sanctuary1 | | 22 | M | 0 | 1460 | 0 | 0.5 | MIXED |
| D | Sanctuary1 | | 13 | F | 312 | 1148 | 0 | 0.393 | MIXED |
| E | Sanctuary1 | | 12 | F | 312 | 1148 | 0 | 0.393 | MIXED |
| A | Sanctuary2 | | 15 | F | 365 | 1095 | 0 | 0.375 | MIXED |
| B | Sanctuary2 | | 15 | F | 0 | 1460 | 0 | 0.5 | MIXED |
| C | Sanctuary2 | | 14 | F | 1460 | 0 | 0 | 0 | HUMAN |
| D | Sanctuary2 | | 31 | F | 0 | 1460 | 0 | 0.5 | MIXED |
| E | Sanctuary2 | | 43 | F | 1460 | 0 | 0 | 0 | HUMAN |
| F | Sanctuary2 | | 15 | F | 0 | 1460 | 0 | 0.5 | MIXED |
| G | Sanctuary2 | | 16 | M | 0 | 1460 | 0 | 0.5 | MIXED |
| H | Sanctuary2 | | 16 | M | 0 | 1460 | 0 | 0.5 | MIXED |
| I | Sanctuary2 | | 18 | F | 0 | 1460 | 0 | 0.5 | MIXED |
| J | Sanctuary2 | | 26 | M | 0 | 1460 | 0 | 0.5 | MIXED |
| K | Sanctuary2 | | 20 | M | 1460 | 0 | 0 | 0 | HUMAN |
| L | Sanctuary2 | | 14 | F | 0 | 1460 | 0 | 0.5 | MIXED |
| A | Sanctuary3 | | 11 | F | 0 | 1460 | 0 | 0.5 | MIXED |
| B | Sanctuary3 | | 10 | F | 912 | 548 | 0 | 0.188 | HUMAN |
| C | Sanctuary3 | | 26 | M | 1460 | 0 | 0 | 0 | HUMAN |
| D | Sanctuary3 | | 11 | M | 548 | 912 | 0 | 0.313 | MIXED |
| E | Sanctuary3 | | 22 | F | 0 | 1095 | 365 | 0.625 | MIXED |
| F | Sanctuary3 | | 30 | F | 365 | 1095 | 0 | 0.375 | MIXED |
| G | Sanctuary3 | | 26 | F | 0 | 1095 | 365 | 0.625 | MIXED |
| A | Zoo1 | | 41 | F | 1460 | 0 | 0 | 0 | HUMAN |
| B | Zoo1 | | 39 | M | 0 | 1460 | 0 | 0.5 | MIXED |
| C | Zoo1 | | 13 | F | 0 | 0 | 1460 | 1 | CHIMP |
| D | Zoo1 | | 17 | F | 0 | 0 | 1460 | 1 | CHIMP |
| E | Zoo1 | | 15 | F | 0 | 0 | 1460 | 1 | CHIMP |
| F | Zoo1 | | 7 | F | 0 | 0 | 1460 | 1 | CHIMP |
| G | Zoo1 | | 27 | F | 0 | 0 | 1460 | 1 | CHIMP |
| H | Zoo1 | | 9 | M | 0 | 0 | 1460 | 1 | CHIMP |

Table S1 Continued

| A | | Zoo2 | | 54 | | F | | 456 | | 456 | | 548 | 0.467 | MIXED |  |
| --- | --- | --- | --- | --- | --- | --- | --- | --- | --- | --- | --- | --- | --- | --- | --- |
| B | | Zoo2 | | 15 | | M | | 0 | | 0 | | 1460 | 1 | CHIMP |  |
| C | | Zoo2 | | 27 | | F | | 0 | | 0 | | 1460 | 1 | CHIMP |  |
| D | | Zoo2 | | 23 | | M | | 0 | | 0 | | 1460 | | 1 | CHIMP |
| E | | Zoo2 | | 25 | | F | | 0 | | 0 | | 1460 | | 1 | CHIMP |
| F | | Zoo2 | | 23 | | F | | 0 | | 0 | | 1460 | | 1 | CHIMP |
| A | | Zoo3 | | 29 | | F | | 730 | | 730 | | 0 | | 0.250 | HUMAN |
| B | | Zoo3 | | 17 | | F | | 120 | | 420 | | 920 | | 0.774 | CHIMP |
| C | | Zoo3 | | 19 | | M | | 0 | | 1460 | | 0 | | 0.5 | MIXED |
| D | | Zoo3 | | 22 | | M | | 0 | | 1460 | | 0 | | 0.5 | MIXED |
| A | | Zoo4 | | 26 | | F | | 0 | | 1460 | | 0 | | 0.5 | MIXED |
| B | | Zoo4 | | 29 | | M | | 0 | | 1460 | | 0 | | 0.5 | MIXED |
| C | | Zoo4 | | 26 | | F | | 0 | | 1460 | | 0 | | 0.5 | MIXED |
| D | | Zoo4 | | 8 | | M | | 0 | | 1460 | | 0 | | 0.5 | MIXED |
| E | | Zoo4 | | 25 | | F | | 0 | | 1460 | | 0 | | 0.5 | MIXED |
| F | | Zoo4 | | 40 | | F | | 0 | | 1460 | | 0 | | 0.5 | MIXED |
| G | | Zoo4 | | 34 | | F | | 0 | | 1460 | | 0 | | 0.5 | MIXED |
| H | | Zoo4 | | 19 | | M | | 0 | | 1460 | | 0 | | 0.5 | MIXED |
| A | | Zoo5 | | 18 | | F | | 0 | | 0 | | 1460 | | 1 | CHIMP |
| B | | Zoo5 | | 13 | | F | | 0 | | 0 | | 1460 | | 1 | CHIMP |
| C | | Zoo5 | | 22 | | M | | 0 | | 0 | | 1460 | | 1 | CHIMP |
| D | | Zoo5 | | 22 | | F | | 0 | | 0 | | 1460 | | 1 | CHIMP |
| E | | Zoo5 | | 18 | | F | | 0 | | 0 | | 1460 | | 1 | CHIMP |
| F | | Zoo5 | | 13 | | M | | 0 | | 0 | | 1460 | | 1 | CHIMP |
| A | | Zoo6 | | 13 | | M | | 425 | | 1030 | | 0 | | .353 | MIXED |
| B | | Zoo6 | | 11 | | M | | 0 | | 0 | | 1460 | | 1 | CHIMP |
| C | | Zoo6 | | 29 | | F | | 0 | | 600 | | 860 | | 0.795 | CHIMP |
| D | | Zoo6 | | 11 | | M | | 0 | | 0 | | 1460 | | 1 | CHIMP |
| E | | Zoo6 | | 16 | | F | | 0 | | 1460 | | 0 | | 0.5 | MIXED |
